# The Future of Pharmacy

**Published:** 1920-01-03

**Authors:** 


### The Future of Pharmacy.

SIR W. GLYN-JONES' WARNING.

At the recent inaugural meeting of the winter session of the Public Pharmacists' Association, held at 17 Bloomsbury Square, Mr. T. H. W. Idris, J.P., took the chair, and there was a good attendance of members.

The following new members were elected: Mr. T. Sefton Bowers, R.N. Hospital, Hong Kong; Mr. W. J. Stevenson, Belfast Infirmary; Mr. L. Billington, Islington Infirmary; Mr. J. D. Robinson, Camberwell Dispensary.

The inaugural address was delivered by Sir William Glyn-Jones, Secretary and Registrar of the Pharmaceutical Society, who asked that he should be allowed to substitute a confidential talk on the affairs of pharmacy for the usually expected formal address. Sir Wm. Glyn-Jones outlined the probability of vast changes in the practice of pharmacy under the Ministry of Health, and suggested that hospital and institutional pharmacists would be obliged to adapt themselves to the new requirements. Sera, vaccines, the preparation of medicaments in the newer forms for administration would all require skill and knowledge, which necessitated pharmacists keeping their information up to date.

Reference was made to the new curriculum now imposed for the qualification of pharmacist, which should tend to raise the professional status of persons holding the statutory qualification.

Messrs. Fouracre, Gibson, Lindsey, and C. T. Holloway took part in the discussion which ensued, and many interesting questions were asked, and answered by Sir W. Glyn-Jones. The general meetings of this Association were suspended during the war, and this meeting will, it is hoped, mark the advent of a new period of usefulness.

